# Missed HIV prevention opportunities: the PrEP cascade among pregnant or parenting adolescent girls and young women in South Africa

**DOI:** 10.3389/frph.2025.1648786

**Published:** 2025-10-17

**Authors:** Jenny Chen-Charles, Linda-Gail Bekker, Janina Jochim, Camille Wittesaele, Lucie Cluver, Elona Toska

**Affiliations:** ^1^ Department of Social Policy and Intervention, University of Oxford, Oxford, United Kingdom; ^2^ Desmond Tutu HIV Centre, University of Cape Town, Cape Town, South Africa; ^3^ MRC International Statistics and Epidemiology Group, Department of Infectious Disease Epidemiology, London School of Hygiene & Tropical Medicine, London, United Kingdom; ^4^ Centre for Social Science Research, Department of Sociology, University of Cape Town, Cape Town, South Africa; ^5^ Department of Psychiatry, University of Cape Town, Cape Town, South Africa

**Keywords:** pre-exposure prophylaxis, PrEP cascade, adolescent girls and young women, pregnant women, parenting women, HIV prevention, sub-Saharan Africa

## Abstract

**Introduction:**

Pregnant or parenting adolescent girls and young women (PPYW) are at greater risk of sexual exposure to HIV than their peers, yet tailored HIV prevention efforts for PPYW remain limited.

**Methods:**

We analysed cross-sectional data (2020–2023) from a sample of PPYW (median age 21.5, IQR = 20.3–22.5) in the Eastern Cape, South Africa.

**Results:**

Approximately 88% of PPYW who were HIV-negative (*n* = 646) had a HIV test in the last few years. Of these—58% knew about PrEP (*n* = 328). Of those who knew about PrEP, 31% had ever been offered PrEP (*n* = 100; 15% of HIV-negative PPYW), and 62% of those who were offered PrEP had ever taken PrEP (*n* = 62; 10% of HIV-negative PPYW). Compared to PPYW who have not had a HIV test in the last few years, PPYW who had accessed HIV testing were more likely to be aware of PrEP (aOR = 2.39, 95% CI:1.44–3.97, *p* = 0.001), have been offered PrEP (aOR = 2.96, 95% CI:1.16–7.55, *p* = 0.023), and taken PrEP (aOR = 4.57, 95% CI:1.09–19.16, *p* = 0.038).

**Conclusions:**

This study highlights missed opportunities in PrEP delivery and offers recommendations to enhance PrEP awareness and uptake among PPYW. Despite high HIV testing rates in this key population, awareness of PrEP, its offer, and uptake remain low.

## 1 Introduction

Pregnant or parenting adolescent girls and young women (PPYW) face a heightened risk of acquiring HIV due to a mix of biological vulnerability and social and structural inequalities (1–3). This group is a key priority for HIV prevention in Eastern and Southern Africa, where they continue to carry a disproportionate share of new infections. In 2023, adolescent girls and young women (AGYW) were three times more likely to acquire HIV compared to males of the same age (4). South Africa remains at the epicentre of the epidemic, with close to eight million people living with HIV (5). Oral pre-exposure prophylaxis (PrEP) offers strong protection against HIV when taken consistently and plays an important role in preventing mother-to-child transmission during pregnancy for women who are HIV-negative (6).

Since its introduction in South Africa in 2016, oral PrEP has been a key component of the country's HIV prevention strategy (7). PrEP is safe to use during pregnancy and breastfeeding, and in 2020, South Africa updated its national guidelines to include pregnant and breastfeeding women (PBW) who test negative on routine HIV testing in the eligibility criteria for PrEP (8–10). Moreover, the guidelines identify PBW as a key population at heightened risk of HIV exposure, and stipulate that they should receive counselling and be offered HIV prevention measures, including PrEP (7). The policy shift recognised the vulnerabilities faced by PPYW, highlighting the importance of scaling up HIV prevention within this group (7). Recent research has also highlighted the importance of understanding PrEP uptake and use among pregnant and breastfeeding women, a high-priority population for HIV prevention (11–13). The 2022 South Africa Antenatal HIV Sentinel Survey reported low levels of PrEP awareness and coverage among pregnant women, highlighting the need to better understand gaps in PrEP awareness and uptake among this population (14). But much of the research to date has centred on adult populations, with limited attention given to the specific experiences and needs of young women, especially PPYW.

Studies from Eastern and Southern Africa suggest that PPYW face a higher likelihood of living with HIV than their peers who have not had children (15, 16). This increased risk is shaped by a range of factors, including relationships with older partners, experiences of transactional sex, and irregular or infrequent condom use. Many only begin using contraception after their first pregnancy and often rely on methods like injectables or oral contraceptives, which offer no protection against HIV (15). As a result, they remain vulnerable to HIV infection during earlier and subsequent periods of sexual activity (17, 18). Furthermore, PPYW often face exacerbated challenges, including school drop-out, unemployment, poverty, social isolation, loss of social support, stigma, and negative experiences at health facilities, all of which act as barriers to PrEP access and uptake (19–25). Limited exposure to comprehensive sexual education due to school drop-out could also impact HIV prevention awareness among PPYW (26). Yet PPYW are under-researched in HIV prevention, especially regarding PrEP.

A scoping review examining factors influencing PrEP uptake and continuation among AGYW identified several key barriers, including low awareness of PrEP, limited access to health services, and negative experiences with clinic staff (27). Furthermore, fears related to potential effects on their foetus during pregnancy and navigating the postpartum period poses additional challenges for PPYW, further highlighting this population to be a priority population for PrEP interventions (27). Evidence also suggests that sociodemographic factors, including younger age and lower socioeconomic status, significantly influence PrEP awareness, uptake and continuation among AGYW (28–30). Understanding how these factors affect PrEP uptake is key to developing effective, context-specific interventions to increase PrEP utilisation among PPYW.

This study used a PrEP cascade framework to investigate awareness, reach, and uptake among PPYW in a PrEP-naïve community. The PrEP cascade is used as an implementation science tool to identify gaps in the delivery of HIV prevention services, particularly among underserved populations (31). Applying this framework enables a stepwise analysis of where and why PPYW may be lost along the PrEP pathway—from HIV testing to PrEP awareness, offer, and uptake. offering insights into where interventions may be needed to better reach PPYW. To our knowledge, this is one of the first studies to apply a PrEP cascade specifically among PPYW, a group recognised as being at very high risk for HIV but often overlooked in prevention research. Data were collected between 2020 and 2023, soon after the PrEP guidelines in South Africa was extended to pregnant and breastfeeding women. Despite this policy change, PrEP was rarely offered to PPYW in our study, underscoring persistent barriers in service delivery and signalling missed prevention opportunities at a critical stage of programme expansion.

## 2 Methods

### 2.1 Study design and participants

This study is a cross-sectional analysis of longitudinal cohort, which surveyed 986 PPYW (including those living with HIV) in the Eastern Cape, South Africa between December 2020 and March 2023. The baseline data collection occurred between 2018 and 2019. Participants were recruited through a combination of strategies including: through an existing study, schools, door-to-door, malls, referrals, and through public health-care facilities [more detail on the recruitment strategy is described elsewhere (17)]. The study took place in a peri-urban and rural health district of the Eastern Cape province, which had the second highest antenatal HIV prevalence rates in South Africa at 32.9% in 2022 (32).

In 2020–2023, 87% of participants who participated at baseline (2018–2019) were re-interviewed, with 2% refusing (*n* = 24), 10% lost to follow-up (*n* = 113), and 1% passed away (*n* = 9). All eligible participants who completed follow-up interviews were included in this study; missing data were minimal and participants with missing values for key measures were excluded from analysis. This study utilised cross-sectional data from the most recent follow-up, as PrEP-related information was not collected at baseline (2018–2019). This reflects the timing of the inclusion of pregnant and breastfeeding women in national PrEP rollout in South Africa, which began in 2020. All participants in this cohort were recruited as mothers under 24 years of age at baseline. As this analysis uses follow-up data, some participants had aged beyond 24 by the time of data collection, resulting in an upper age limit of 28. Including these participants allows us to capture the experiences of young mothers as they transition into later young adulthood.

### 2.2 Data collection

Trained research assistants conducted telephone interviews with participants during remote data collection activities necessitated by COVID-19 lockdown restrictions, which began at the end of March in 2020 and ended in June 2022. Although data collection continued until March 2023, we continued using remote methods to ensure participant safety and study continuity. Structured questionnaires were administered in participants' preferred language (isiXhosa or English) (33). Questionnaires were translated from English into isiXhosa, and then back-translated into English to ensure accuracy and cultural appropriateness. The full questionnaires for the study are available at https://www.heybaby.org.za/research, and the measures used in this analysis can be found in Supplementary Appendix S1. The questionnaire was developed using validated measures that had been previously applied in South Africa where possible. For constructs without existing local measures, items were developed by the study team, drawing on prior literature and tailored to the context of the participants. Data collection tools were piloted with a subset of participants to ensure clarity and appropriateness (25).

### 2.3 Ethical considerations

The study received ethical approval from the Universities of Oxford (CUREC ref: R48876/RE003) and Cape Town (HREC ref: 226/2017; 027/2024), and local governmental and provincial bodies. Informed consent was obtained from all participants, and from their caregivers for participants who were <18 years old. Measures were taken to ensure their privacy and confidentiality throughout the study. Participants were not given any financial incentives but received airtime/data worth R30.00 as a token of appreciation.

### 2.4 Measures

The primary outcome for this analysis is the PrEP cascade, adapted from HIV prevention cascades in existing literature (34). In this study, each step was measured using binary variables. The PrEP cascade begins by assessing whether participants had accessed HIV testing since their baseline interview (2017–2019), with an average interval of three years between interviews, as HIV testing is expected to facilitate subsequent PrEP offer in accordance with South African guidelines (7). We then assessed awareness of PrEP (HIV-negative participants were asked “*Do you know about PrEP?*” after a brief introduction and explanation of PrEP—yes vs. no). This was followed by whether participants were ever offered PrEP (only those who responded that they knew PrEP answered: “*Have you ever been offered PrEP?*”—yes vs. no), and finally whether they had ever taken PrEP when offered (only those who said they were offered PrEP answered: “*Have you ever taken PrEP?*”—yes vs. no).

Binary variables were created from survey questions. Sociodemographic factors include age (<20 years old vs. ≥20 years old), and age at first childbirth (<20 years old vs. ≥20 years old). The <20 years age cut-off was selected based on national definitions of adolescent childbearing used in South African demographic and health research (35). Other factors include multiple parity (≥2 children vs. only one child), enrolled in school or in employment (vs. not enrolled in school or employed), type of housing (formal vs. informal), household poverty [ability to afford the eight highest socially-perceived necessities, based on South African survey measures—including school fees, school uniform, school supplies, shoes, clothes—vs. lack of access (35)], and food insecurity (at least one day without enough food at home in the past week vs. enough food).

Factors on clinic experiences were also included to identify whether clinic experiences impacted PrEP uptake, as seen in other studies identified in a scoping review (27). These were measured through perceived interactions with clinic staff using binary variables: staff never displaying anger, clinic staff always having time to provide necessary assistance, and participants' confidence that their information would be kept safe and confidential. All measures were self-reported (details on all measures can be found in Supplementary Appendix S1).

### 2.5 Statistical analysis

First, descriptive analyses were conducted to examine the relationship between one's history of HIV testing since baseline with socio-demographic variables and the PrEP cascade. Participants who had not tested for HIV were retained in the denominator at each step of the cascade, ensuring that their awareness, offer, and uptake of PrEP were captured and directly comparable to those who had tested. Secondly, multivariable logistic regression was used to evaluate the association between HIV testing and the PrEP cascade steps (awareness, offer, and uptake), controlling for sociodemographic variables including age, age at first childbirth, multiple parity, enrolled in school or working, type of housing, household poverty, and food insecurity. Adjusted odds ratios (aORs) with 95% confidence intervals (CIs) are reported to assess the strength of these associations. Statistical significance was defined as *p* < 0.05.

Finally, stepwise multivariable regression models were used to identify factors significantly associated with PrEP awareness. For the smaller samples of PrEP offered (*n* = 100) and PrEP taken (*n* = 62), stepwise regression was deemed inappropriate due to the increased risk of overfitting and unreliable parameter estimates. We selected variables for the models following Hosmer and Lemeshow's purposeful selection of variables, based on theoretical considerations from existing literature (36). In the first model, we applied forward selection and included all sociodemographic factors, HIV testing, and clinic experience variables hypothesised as potential covariates. The second model included only factors that were significant at a 10% level (*p* < 0.1). The third and final model included only factors significant at the 5% level (*p* < 0.05). The goodness-of-fit of the logistic regression model was assessed using the Hosmer-Lemeshow test (36). All analysis was conducted using STATA17 (37).

## 3 Results

The study included *n* = 646 HIV-seronegative PPYW, aged 16–28 years (median age 21.5, IQR = 20.3–22.5). One in five (20%, *n* = 130) were <20 years old, and the majority (96%, *n* = 604) had their first child before age 20. Only 2% were pregnant at the time of data collection. Almost one in four (23%, *n* = 149) had two or more children, and 32% (*n* = 206) lived in informal housing. Only 13% (*n* = 83) lived with no poverty (household could afford all basic necessities), and 36% (*n* = 234) did not experience food insecurity in the past week. More than half (55%, *n* = 355) were either in school/university or employed (see Table 1).

**Table 1:** Sample characteristics of pregnant or parenting adolescent girls and young women in the Eastern Cape, South Africa (2020–2023).

Characteristic	Total *N* = 646	HIV testing since baseline *N* = 566 (88%)	No HIV testing since baseline *N* = 80 (12%)	*p-value*
Median age = 21.5 (IQR = 20.3–22.5)				
Age <20	130 (20%)	105 (85%)	18 (15%)	0.390
Age at birth of first child <20	604 (96%)	523 (87%)	75 (13%)	0.256
Multiple children (≥2)	149 (23%)	132 (92%)	12 (8%)	0.093
Informal housing	206 (32%)	182 (91%)	17 (9%)	*0.044
No poverty—household can afford all necessities	83 (13%)	65 (82%)	14 (18%)	0.120
Food insecurity	234 (36%)	208 (89%)	26 (11%)	0.459
In education or employment	355 (55%)	300 (90%)	33 (10%)	*0.044
Clinic experience variables				
Clinic experience—staff never angry	399 (62%)	363 (91%)	36 (9%)	*0.001
Clinic experience—staff never too busy	364 (56%)	308 (91%)	30 (9%)	*0.003
Clinic experience—information kept confidential	372 (58%)	330 (91%)	34 (9%)	*0.008

### 3.1 The PrEP cascade

**Figure 1:**
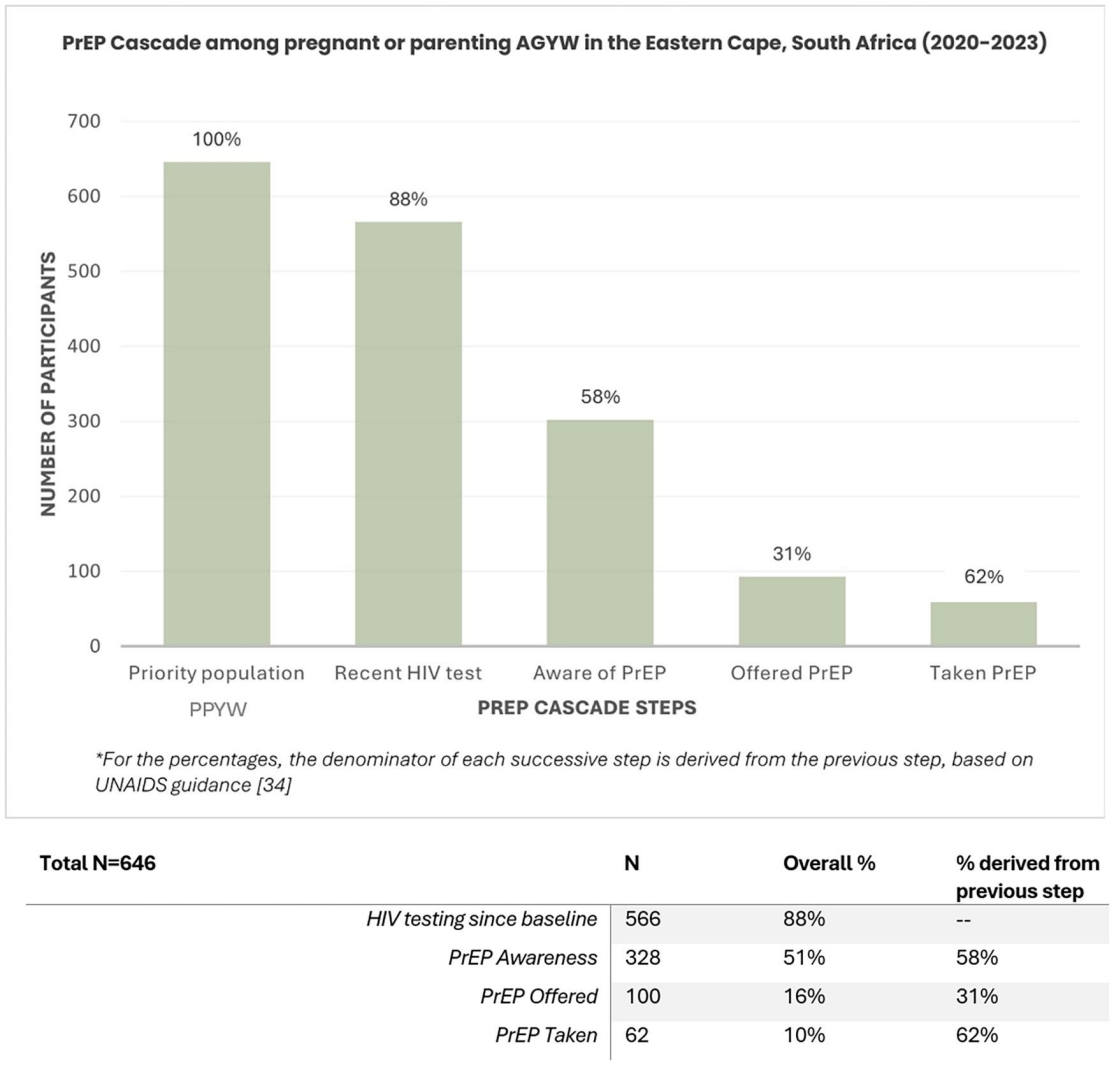
PrEP Cascade among pregnant or parenting adolescent girls and young women in Eastern Cape, South Africa (2020-2023).

Among those surveyed, 88% (*n* = 566) reporting having had a HIV test since baseline. However, cascade progression dropped steeply at each step: only 58% of those tested (*n* = 328) were aware of PrEP; among those aware, only 31% (*n* = 100) had ever been offered PrEP; and among those offered PrEP, 62% (*n* = 62) had ever taken PrEP (see Figure 1). Among the 80 participants who did not report having a HIV test since baseline, 33% (*n* = 26) were aware of PrEP, 6% (*n* = 5) had been offered PrEP, and 3% (*n* = 2) reported ever initiating PrEP.

In adjusted multivariable regression models, HIV testing was significantly associated with advancing through all stages of the PrEP cascade among PPYW, after adjusting for age, parity, school enrolment or employment, poverty status, food insecurity, and housing type. PPYW who had accessed HIV testing since baseline were two times more likely to be aware of PrEP (aOR = 2.39, 95% CI: 1.44–3.97, *p* = 0.001). HIV testing was also significantly associated with higher odds of PPYW being offered PrEP (aOR = 2.96, 95% CI: 1.16–7.55, *p* = 0.023), and with higher rates of PrEP uptake (aOR = 4.57, 95% CI: 1.09–19.16, *p* = 0.038) (see Table 2).

**Table 2:** Multivariable regression results for associations between HIV testing since baseline and prEP awareness, offer, and uptake among pregnant or parenting adolescent girls and young women in the eastern cape, South Africa (2020–2023).

HIV testing since baseline (*n* = 646)	aOR*	95% CI	*p*-value
PrEP Awareness	2.38	1.43–3.96	0.001
PrEP Offered	2.98	1.16–7.64	0.023
PrEP Taken	4.55	1.08–19.14	0.038

* Adjusted for age, age at birth of first child, parity, school enrolment or employment, poverty status, food insecurity, and type of housing.

### 3.2 Factors associated with PrEP awareness in stepwise regression

The Hosmer-Lemeshow test indicated a satisfactory fit with a chi-square statistic of 0.06 and a *p*-value of 0.9715, suggesting that the model adequately represents the observed data (36). The final model of the stepwise regression indicates a few significant predictors of PrEP awareness among participants (see Table 3). School enrolment/being employed was the only sociodemographic variable that was significantly associated with increased PrEP awareness (aOR = 1.66; 95% CI: 1.21–2.27, *p* = 0.002). HIV testing since baseline remained significantly associated with increased PrEP awareness (aOR = 2.13; 95% CI: 1.29–3.53, *p* = 0.003). Positive clinic experience as reported by absence of anger from clinic staff was also significantly associated with greater PrEP awareness (OR = 1.46; 95% CI: 1.05–2.03, *p* = 0.023).

**Table 3:** Factors associated with PrEP awareness among pregnant or parenting adolescent girls and young women in the Eastern Cape, South Africa (2020–2023).

Total *N* = 646	Model 1	Model 2	Model 3
	aOR (95% CI)	aOR (95% CI)	aOR (95% CI)
Age (<20)	*0.70 (0.46–1.04)	–	–
Age at birth of first child (<20)	0.90 (0.39–2.09)	–	–
Multiple children	0.79 (0.55–1.17)		
Enrolled in school or employed	*1.65 (1.19–2.28)	*1.66 (1.21–2.27)	**1.66 (1.21–2.27)** ***p*** ***=*** ***0.002***
Informal home	0.82 (0.58–1.17)	–	–
Afford all necessities	1.46 (0.89–2.40)	–	–
HIV testing since baseline	*2.30 (1.38–3.86)	*2.13 (1.29–3.53)	**2.13 (1.29–3.53)** ***p*** ***=*** ***0.003***
Clinic experience—staff never angry	*1.69 (1.03–2.78)	*1.46 (1.05–2.03)	**1.46 (1.05–2.03)** ***p*** ***=*** ***0.023***
Clinic experience—staff never too busy	1.10 (0.70–1.73)	–	–
Clinic experience—information kept confidential	0.77 (0.48–1.24)	–	–
	*Significant at the 0.10 level	*Significant at the 0.05 level	

Bold values indicate significant at the 0.05 level.

## 4 Discussion

Using a PrEP cascade framework, our findings show gaps in PrEP awareness, offer, and uptake among PPYW in South Africa, a population at heightened risk of HIV acquisition, but insufficiently reached by existing HIV prevention programmes. This study was conducted in a community with limited prior exposure to PrEP; there were no widespread PrEP programmes in place at the time of data collection. Therefore, the low levels of awareness and offer observed are likely reflective of broader systemic issues in introducing and scaling up PrEP in settings where it has not yet been meaningfully implemented. Even among PPYW who were recently in contact with the healthcare system, including HIV testing, only half were aware of PrEP and fewer than one in five had ever been offered it. Yet, among those offered PrEP, nearly two-thirds reported initiating it, suggesting unmet demand and underutilised opportunities within existing service delivery platforms—aligning with findings from previous studies (27).

These findings show that there are critical gaps in the implementation of PrEP within routine care and the missed opportunities to engage this key population through existing health system touchpoints. Our results align with recent guidance from AVAC, which emphasises the need for youth-focused PrEP delivery in South Africa and other countries in the region and highlighting the importance of closing implementation gaps to reach adolescent and young populations effectively (38). Similar gaps in PrEP cascades and barriers to PrEP access have been observed in studies, including DREAMS, conducted in other sub-Saharan African countries such as Kenya, Uganda and Zimbabwe, as synthesised in our recent scoping review (25). This suggest that these gaps are not unique to PPYW in South Africa, highlighting the need for regional strategies to strengthen service delivery and expanding equitable access.

Our findings indicate that the primary gap in the PrEP cascade is between HIV testing and PrEP offer. While most participants had recently had HIV testing, the majority had not been offered PrEP. Among PPYW who were offered PrEP, approximately two-thirds accepted it. Although we did not directly ask participants whether they interested in taking PrEP, the observed pattern suggest that low uptake may not be due to lack of interest but because of limited access, consistent with findings from studies. Previous research has highlighted potential provider or system-level barriers, such as inconsistent risk assessments, limited provider capacity or confidence, or lack of adherence to PrEP guidelines (27, 31, 39, 40). Although South African PrEP guidelines recommend offer to individuals at substantial risk, it remains unclear whether PPYW are being systematically assessed or categorised as eligible.

PrEP should be made available and accessible in services that are already accessed by PPYW, including sexual reproductive health, family planning, and child health clinics. This requires not only policy alignment but also the allocation of resources, training, and support to overcome existing barriers to integration (41, 42). Looking ahead, as long-acting PrEP modalities such as the dapivirine vaginal ring and injectables, i.e., Cabotegravir (bi-monthly) and Lenacapavir (twice-yearly), becomes more widely available, effective integration would also be crucial to maximise their uptake and adherence among PPYW—a key population that could benefit significantly from long-acting PrEP (43–45). Given the low levels of PrEP offer observed in our cascade, ensuring that PPYW are informed about and offered long-acting PrEP modalities will be critical to maximising uptake and adherence in this underserved population and to closing gaps in the PrEP cascade.

In our study, PPYW who were not engaged in school or work were less likely to be aware of PrEP. This suggests that they may be more socially and economically marginalised, with fewer opportunities for health education and service uptake. Previous research has identified pregnancy and early motherhood as major drivers of school dropout among AGYW in South Africa, and few return to finish their education, limiting future employment opportunities (22–24). The DREAMS PrEP Choice Study in South Africa found that most participants received services from educational and training institutions (46). These findings highlight the importance of targeted outreach strategies beyond formal institutions to reach PPYW who are structurally and socially marginalised. Addressing barriers like inflexible health services, limited access to information platforms, as well as low educational attainment and poverty, may help to increase PrEP adherence among these underserved young women (47, 48).

In addition, PPYW are often stigmatised and face judgemental attitudes from health care workers due to being young mothers, which is a well-documented barrier to healthcare (19, 20). Not experiencing anger from clinic staff when discussing sensitive topics was associated with higher PrEP awareness. This finding reinforces the critical role of a supportive, non-judgmental healthcare environment in promoting PrEP awareness for young women, as shown in other studies (27).

### 4.1 Limitations

The cross-sectional approach does not allow for causal associations to be established between predictors and PrEP cascade outcomes, and we cannot determine the directionality of associations. The possibility for reversed associations cannot be ruled out (e.g., whether increased PrEP awareness leads to increased HIV testing) or the potential effects of confounding factors on observed associations. The reliance on self-reported data for HIV testing, PrEP awareness, and uptake may have introduced bias because clinic records were not obtained. PrEP awareness was measured using a single binary question, which does not capture participants' depth of knowledge, including understanding of side effects or adherence requirements. Participants may have been likely to underreport their use of PrEP due to stigma around disclosing sexual activity and community stigma around PrEP due to its connection with HIV, potentially leading to an underestimation of PrEP coverage in the study findings. While validated measures were used where possible, some questionnaire items were adapted or developed for this study; however, they were piloted with a subset of participants to ensure clarity and relevance. We also could not conduct stepwise regression with PrEP offer and uptake due to the small sample sizes for the two variables. In addition, although uptake among participants who were offered PrEP was relatively high, our data cannot confirm whether this is due to selective offering by providers.

While recruitment strategies focused on creating a representative sample of PPYW through health facility- and community-based recruitment, findings may not be generalisable to PPYW in different regions of South Africa or other countries with distinct cultural, economic, or healthcare contexts. This study was conducted during the period of South Africa's COVID-19 lockdowns and the period of recovery. As such, it offers valuable insight into how PrEP access and uptake among PPYW were shaped during a time of significant disruption as well as in the months that followed. We also did not collect data on PrEP persistence, which would provide valuable insights for this population in future research. We also did not collect data on PrEP awareness among participants who reported living with HIV; examining this in future research could provide valuable insights.

## 5 Conclusions

This study showed how, despite their elevated HIV acquisition risk, PPYW in South Africa continue to be left behind in HIV prevention programmes. In communities where PrEP has not been introduced through implementation programmes, awareness is low, and opportunities to deliver it during routine healthcare are missed. Our findings highlight the fact that many participants had recent contact with health services but were not informed of or offered PrEP, indicating systemic inadequacies rather than a lack of demand. Improving access means more than just making PrEP available—it needs to be integrated into the services PPYW already use, such as antenatal clinics, child health visits, and sexual and reproductive healthcare. However, for this to work, health workers need to receive practical support and training, and clinics must provide a safe, non-judgmental environment for young women.

At the same time, broader challenges that PPYW face need to be considered. Barriers such as dropping out of education early and limited employment opportunities all influence how and whether young moms may seek or keep healthcare. Tackling these concerns in conjunction with service delivery improvements will be critical to ensure that PPYW does not fall behind. Being out of school or unemployed can cut people off from information and can make clinic visits difficult. Policies and services that support education, employment, and parenting could make a meaningful difference in their ability to engage with healthcare. As longer-acting PrEP options become available, these findings offer practical insights into how programmes can better meet the needs of young pregnant or parenting women. PPYW are often left out of HIV prevention strategies. Reaching them with the right tools, delivered in the right ways, is essential to reducing new infections in this priority population.

## Data Availability

The data that support the findings of this study are available from the corresponding author upon reasonable request. The data are not publicly available due to privacy or ethical restrictions. Requests to access the datasets should be directed to https://static1.squarespace.com/static/5ef06d788b06a83de4933740/t/64941fdf78be1d23e279538a/1687429087463/20190513_DataUseUndertaking_MASTER.pdf.
