# Supplementary material for: Missed HIV prevention opportunities: the PrEP cascade among pregnant or parenting adolescent girls and young women in South Africa
Source: Front Reprod Health. 2025 Oct 17;7:1648786. doi: 10.3389/frph.2025.1648786 (PMC12575254; doi:10.3389/frph.2025.1648786)
Supplement: Supplementary file 1 [file Table1.docx]

| **Variable** | **Type of Data and Definition** |
| --- | --- |
| ***PrEP Cascade measures***  *Developed and piloted with adolescent mothers* | |
| HIV testing | Dichotomised responses to: ‘Since we last saw you, have you had an HIV test?’ |
| PrEP Knowledge | *Introduction: We would like to ask you about PrEP. PrEP is a pill taken regularly by someone who is not living with HIV*  *to prevent them from getting HIV. PrEP needs to be taken for seven days before sex to be effective.*  Dichotomised items to: ‘Do you know about PrEP?’ [1=Knows PrEP, 0 = Does not know PrEP], based on responses 1) I know about PrEP already – I know how it works, 2) I have heard about PrEP but not sure how it works, 3) I did not know about this |
| Offered PrEP | Dichotomised responses to: ‘Have you ever been offered PrEP’ |
| Taken PrEP | Dichotomised responses to ‘Have you ever taken PrEP’ |
| ***Sociodemographic measures*** | |
| Age  *Measured using item from the South African Census* | Dichotomised as <20 years old or ≥20 years old |
| Age at first childbirth  *Measured using item from the South African Census* | Dichotomised as <20 years old or ≥20 years old when they gave birth to their first child |
| Multiple parity  *Measured using item from the South African Census* | Dichotomised as if they have multiple children (≥2) or not |
| Enrolled in school or working  *Measures were developed with the South African Department of Education and used in a randomised controlled trial conducted among adolescent parents in the Eastern Cape, South Africa (Lester, 2015).* | Dichotomised as if they are enrolled in school/employed or not |
| Type of housing  *Measured using an item from the South African Census* | Dichotomised: Formal or informal (shack) housing |
| Household poverty  *Poverty was measured using items from the South African* | Dichotomised: If they have/do not have access to all top eight essential needs for children and adolescents as defined by a South African Social Survey, which includes adequate warm clothing, shoes, three meals a day, school supplies, school uniform, school fees, handwashing facilities, and toiletries. |
| Food insecurity  *Measured using an item from the South African National Food Consumption Survey* | Dichotomised: If they have/do not have consistent availability of sufficient food at home over the past week |
| ***Clinic experiences measures***  *Measured using items developed and included in the baseline questionnaire, which were piloted with members of the Teenage Advisory Group (Toska et al., 2022)* | |
| Clinic staff showing anger | Dichotomised to: never experiencing clinic staff getting angry vs experiencing anger from clinic staff for having sex |
| Clinic staff too busy | Dichotomised to: clinic staff never too busy to give the help they need vs not always experiencing clinic staff to busy to give them the help they needed |
| Clinic confidentiality | Dichotomised to: always feeling clinic would keep their information confidential and safe vs not always feeling clinic would keep their information confidential or safe |

**References**

Lester, S. N. (2015). *Evaluation of the Parent Centre’s positive parenting skills training programme: a randomised controlled trial* [University of Cape Town]. http://hdl.handle.net/11427/15615

Toska, E., Saal, W., Chen Charles, J., Wittesaele, C., Langwenya, N., Jochim, J., Steventon Roberts, K. J., Anquandah, J., Banougnin, B. H., Laurenzi, C., Sherr, L., & Cluver, L. (2022). Achieving the health and well-being Sustainable Development Goals among adolescent mothers and their children in South Africa: Cross-sectional analyses of a community-based mixed HIV-status cohort. *PLOS ONE*, *17*(12), e0278163. https://doi.org/10.1371/JOURNAL.PONE.0278163
